# Has information technology finally been adopted in Flemish intensive care units?

**DOI:** 10.1186/1472-6947-10-62

**Published:** 2010-10-19

**Authors:** Kirsten Colpaert, Sem Vanbelleghem, Christian Danneels, Dominique Benoit, Kristof Steurbaut, Sofie Van Hoecke, Filip De Turck, Johan Decruyenaere

**Affiliations:** 1 Department of Intensive Care, Ghent University Hospital, Ghent, Belgium; 2 Dpt. of Nursing Staff, Ghent University Hospital, Ghent, Belgium; 3 Dpt. of Information Technology, Ghent University, Ghent, Belgium

## Abstract

**Background:**

Information technology (IT) may improve the quality, safety and efficiency of medicine, and is especially useful in intensive Care Units (ICUs) as these are extremely data-rich environments with round-the-clock changing parameters. However, data regarding the implementation rates of IT in ICUs are scarce, and restricted to non-European countries. The current paper aims to provide relevant information regarding implementation of IT in Flemish ICU's (Flanders, Belgium).

**Methods:**

The current study is based on two separate but complementary surveys conducted in the region of Flanders (Belgium): a written questionnaire in 2005 followed by a telephone survey in October 2008. We have evaluated the actual health IT adoption rate, as well as its evolution over a 3-year time frame. In addition, we documented the main benefits and obstacles for taking the decision to implement an Intensive Care Information System (ICIS).

**Results:**

Currently, the computerized display of laboratory and radiology results is almost omnipresent in Flemish ICUs, (100% and 93.5%, respectively), but the computerized physician order entry (CPOE) of these examinations is rarely used. Sixty-five % of Flemish ICUs use an electronic patient record, 41.3% use CPOE for medication prescriptions, and 27% use computerized medication administration recording. The implementation rate of a dedicated ICIS has doubled over the last 3 years from 9.3% to 19%, and another 31.7% have plans to implement an ICIS within the next 3 years. Half of the tertiary non-academic hospitals and all university hospitals have implemented an ICIS, general hospitals are lagging behind with 8% implementation, however. The main reasons for postponing ICIS implementation are: (i) the substantial initial investment costs, (ii) integration problems with the hospital information system, (iii) concerns about user-friendly interfaces, (iv) the need for dedicated personnel and (v) the questionable cost-benefit ratio.

**Conclusions:**

Most ICUs in Flanders use hospital IT systems such as computerized laboratory and radiology displays. The adoption rate of ICISs has doubled over the last 3 years but is still surprisingly low, especially in general hospitals. The major reason for not implementing an ICIS is the substantial financial cost, together with the lack of arguments to ensure the cost/benefit.

## Background

Over the past decades there have been substantial changes in medicine, with more effective but also increasingly complex therapies. This results in an increased life expectancy on the one hand, but also in an increased number of medical errors on the other hand. In 2003, the Institute of Medicine published the groundbreaking report "To err is human, building a safer health system". This report estimated that at least 44,000 people die in US hospitals each year as a result of medical errors that could have been prevented [1]. Furthermore, the progress in medicine is at least partially responsible for the increasing health care cost, which has risen exponentially over the last two decades. At present it even comprises between 10 to 16% of the gross domestic product in developed countries. Several organisations claim that Information Technology (IT) could contribute in a significant way to improving the quality of health care while at the same time controlling costs [2]. However, until now, no strong evidence has been provided.

The intensive care unit (ICU) has several typical characteristics which make it favorable for computerization, because caring for the critically ill is even more complex, resulting in substantially higher numbers of medical errors and costs [3,4]. Donchin et al. reports an incidence rate of 1.7 errors per patient per Intensive Care Unit (ICU) day and several other authors have confirmed that the ICU is a very unsafe environment [5-9]. In addition, the cost of intensive care medicine is exorbitant and can be as high as 0.5 to 1% of the gross domestic product [10]. Various US critical care organizations made some recommendations to the government in 2004 in answer to what they called "the critical care medicine crisis". Their second recommendation was that "information technology should be leveraged in critical care to promote standardization and improve efficiency" and that "information technology is a key factor in the future of intensive care medicine delivery" [10-12].

For the above reasons it is advisable to study the current level of intensive care computerization, both for general and dedicated specialized IT applications used in the ICU. General IT applications are the electronic patient record, the computer laboratory system (also known als the Global Laboratory Information Management System (GLIMS)), the computer radiology system (i.e. Picture Archiving and Communication System (PACS)), and the Computerized Physician Order Entry (CPOE) applications. The term CPOE can be confusing however, because some authors restrict its use to prescribing medication, and add the term "order communication system" for laboratory and radiology requests. In this paper, we will use CPOE in the broader sense, and we will specify reference to medication CPOE, laboratory CPOE or radiology CPOE. The dedicated IT solution for the ICU is often described as an ICU Patient Data Management System (PDMS), but we prefer the term "Intensive Care Information System" (ICIS), which describes the broader functionalities of more advanced IT programs better, i.e. doing more than mere data storage and representation. These systems are developed in order to meet the specific requirements to optimize data processing and workflow support in critical care medicine. In our survey, an ICIS has to fulfil all of the following conditions: (i) automated collection of physiological and monitoring variables from monitors and ventilators, (ii) incorporation of CPOE for medication prescription and (iii) one bedside personal computer for every ICU bed.

In this paper, the present IT adoption rate in ICUs in Flanders (Belgium) is evaluated, as well as its evolution within a 3-year time frame. This includes both the use of general hospital information system components such as the electronic patient record, CPOE for medication, radiology and laboratory requests and the computerized display of these results, as well as ICU-specific IT software such as ICIS. Furthermore, we have explored the main benefits and obstacles for taking the decision to implement an ICIS as perceived by ICU directors.

## Methods

This study is based on two separate but complementary surveys conducted in the region of Flanders.

### Survey development

The first survey was performed in January 2005 and consists of a written questionnaire which was sent to the medical directors of all Flemish ICUs. Six weeks later the non-respondents were sent a reminder. After another month the remaining non-respondent ICU directors were contacted by phone, and a second reminder was sent when necessary.

The second survey was carried out in October 2008 (i.e. 3.5 years after the first survey). This telephone survey was carried out by K.C., who interviewed each ICU director, or the ICU head nurse if the medical director suggested that the head nurse was the more competent person regarding IT use in the ICU.

The Local Ethical Committee of Ghent University Hospital approved the study, and informed consent was waived. All answers were kept confidential.

### Region of interest

Flanders has 6,117,440 inhabitants and represents the largest region of the federal state of Belgium. The federal state contains 10,580,000 inhabitants. The Region of Flanders had a total of 54 ICUs in 2005, and a total of 63 ICUs in 2008. Differences between these two numbers are mainly due to changing alliances between hospitals, or new approved ICUs (all part of general hospitals) which have been approved by the government. All these ICUs provide mechanical ventilation and are approved by the national government (as listed on http://www.health.fgov.be). They are located in three different types of hospitals: general hospitals (52/63 or 82.6%), tertiary non-academic referral hospitals (8/63 or 12.7%), and university hospitals (3/63 or 4.7%). General hospitals have approximately 250 to 700 beds, tertiary non-academic referral hospitals 500 to 1,100 beds and university hospitals 700 to 1,600 beds. The number of ICU beds consists of 5 to 7% of the total number of hospital beds (excluding post-anaesthesia care beds, specific coronary care unit beds and neonatology beds). Especially the larger hospitals have 24/7 junior or senior critical care physicians available, whereas smaller ICUs usually have anesthesiologist-intensivists (with board certificate in critical care) who combine their anesthesia care with intensive care.

### Domains of interest

For the construction of the surveys, five domains of interest were selected:

1. **Use of general IT programs within the ICU**, such as the use of the electronic patient record, computerized display of laboratory and radiology results, CPOE for laboratory and radiology requests, CPOE for medication prescription and computerized recording of medication administration;

2. **Use of an ICIS in the ICU**, or the intention to implement an ICIS in the near future;

3. **The level of integration between the available ICIS and the Hospital Information System **such as administrative data exchange, connection to the pharmacy information system for automatic medication ordering and dispensing, and automatic billing;

4. The effective use of highly detailed **data extraction from implemented ICISs**;

5. **The decision-making process **in implementing an ICIS, including recording of the perceived benefits and obstacles by the ICU management decision makers.

### Statistics

Statistical analysis was performed using the SPSS 15 software package. The chi-square test was used to compare proportions. A P-value less than 0.05 was considered statistically significant.

## Results

For the 2005 survey, we obtained 31 responses from the 54 hospitals which had been selected for the study, representing a response rate of 57.4%. Twenty-six of these hospitals (83.9%) were general hospitals, 3 (9.7%) were tertiary non-academic referral hospitals and 2 (6.4%) were university hospitals. The number of ICU beds per ICU varied between 6 and 56.

For the 2008 telephone survey, a 100% response rate was obtained (63/63). It must be stressed that both the 2008 telephone survey and the 2005 questionnaire probed into the use of hospital information system components and the availability of ICIS. For this reason, the results concerning the IT adoption rate, and its evolution over time, are highly accurate. The results regarding the benefits of and obstacles for implementing an ICIS have mainly been derived from the 2005 written questionnaire.

### 1) Use of general IT programs within the ICU (Figure 1)

**Figure 1:**
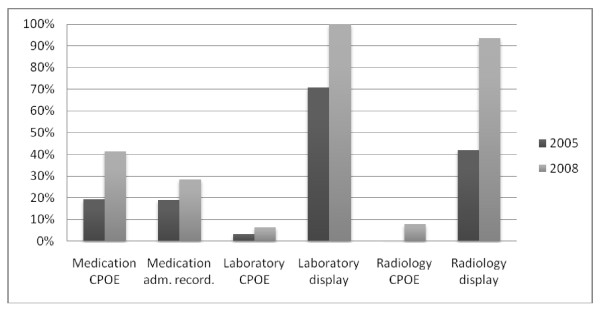
**Implementation and usage rate of general IT components within the ICU**. Medication CPOE = medication prescription by computerized physician order entry; Medication administr. recording = computerized recording of medication administration; Laboratory CPOE = computerized physician order entry of laboratory tests; Laboratory display = computerized display of laboratory results; Radiology CPOE = computerized physician order entry of radiology requests; Radiology display = computerized display of radiology images and/or protocol.

• Electronic patient record

In 2008, 41 out of 63 ICUs (63.1%) use the hospital electronic patient record within the ICU. This is a limited increase compared to 2005 (16/31 ICUs or 51.6%). Of these 31 hospitals, an additional 7 ICUs shifted to an electronic patient record during the 3-year time frame.

• Medication CPOE and the computerized recording of medication administration

A larger proportion of ICUs report using medication CPOE in 2008 compared to 2005: 41.3% vs. 19.3%. Currently, 11 of these CPOE programs are part of an ICIS, 7 have been bought commercially for use in the entire hospital and another 8 CPOE programs are hospital-specific and have been developed in-house. Many of the latter less sophisticated programs needed extensive adaptations, however, especially for continuous infusion pump recording. Another 5 ICUs are using Microsoft Office documenting (i.e. Excel), but as this software has not been developed specifically for medication CPOE we did not include it as such. One ICU that implemented an ICIS more than a decade ago still uses paper-based medication prescriptions, and chose to discard the available CPOE functionality due to integration problems with the pharmacy department. It is important to note that only 6 of the CPOE programs that are not part of a dedicated ICIS also provide facilities for computerized medication administration recording by the nursing staff. In the other ICUs, print-outs of the medication CPOE are taken and used as part of the paper charts.

• The computer laboratory system.

At present, every Flemish ICU uses the computerized display of laboratory results, whereas this was only 70.9% in 2005 (see Figure 1). However, the number of ICUs that use CPOE for laboratory requests is still extremely low (6.3% in 2008 vs. 3.2% in 2005), and half of them does this by using the built-in functionalities of their ICIS.

• The computer radiology system (i.e. The Picture Archiving and Communication System (PACS)).

A larger proportion of ICUs report using the computerized display of radiology results and the CPOE module for radiology requests in 2008 compared to 2005 (93.5% vs. 41.9%; 7.9% vs. 0%) (as presented in Figure 1).

### 2) Use of an ICIS in the ICU, or the intention to implement an ICIS in the near future

In 2008, 12 out of 63 ICUs (19%) had implemented an ICIS and another 20 (31.7%) ICUs were planning to implement a system within the next 3 years (i.e. before 2012), of which 5 were scheduled to go live in 2009.

In 2005, 5 out of 31 (16.1%) of responding hospitals had an ICIS in place. Another 7 hospitals (22.5%) had the intention of implementing an ICIS within the next 3 years (i.e. before 2009), while the remaining 19 hospitals (61.3%) had no explicit intention to implement an ICIS. None of the seven ICUs from the 2005 survey which intended to adopt an ICIS within the following 3 years, actually did so. Finding adequate financing, together with anticipated integration problems with the hospital information system, were the main reasons for postponing the implementation. However, 3 hospitals which showed no interest in adopting an ICIS in 2005, did implement an ICIS before the end of 2008 (cf. Table 1).

**Table 1:** Number of ICIS implementations in 2005-2008

	2005 (n = 54)	2008 (n = 63)
ICIS, n (%)	5 (9.3%)	12 (19.0%)
ICIS uptake <3 years, n (%)	7 (13.0%)	20 (31.7%)
No ICIS, n (%)	42 (77.7%)	31 (49.2%)

ICIS: ICIS already implemented; ICIS uptake <3 yrs: intention of implementing ICIS within the next 3 years; no ICIS: no ICIS available or planned within the first 3 years.

**Figure 2:**
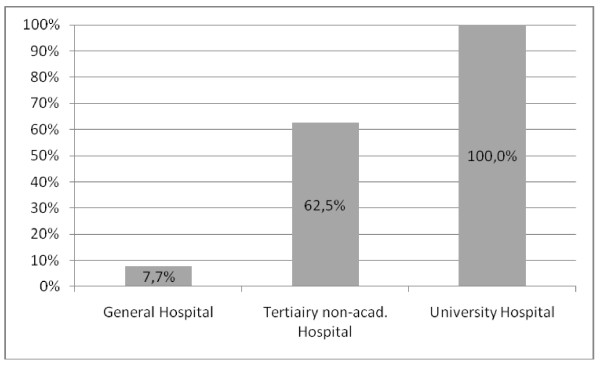
**Implementation rate of ICIS as a function of the type of hospital**. Tertiary non-acad. Hospital: Tertiary non-academic Hospital

There is a significant correlation between the type of hospital and the availability of an ICIS (P < 0.001) (Figure 2). ICUs with ICISs also have a significantly higher number of ICU beds (average 23.9 vs. 11.5 beds).

All ICISs are commercial systems, and the market is shared between a variety of software vendors (as represented in Table 2).

**Table 2:** Implementation rate of different commercial ICISs in Flemish ICUs.

Product	Vendor	n
Centricity™ Critical Care	GE Healthcare IT	3
ICM	Dräger	1
ICIP Critical Care/Care Vue Chart	Philips	3
MetaVision^® ^Clinical Information System	iMD*soft*	3
Picis Critical Care Manager	Picis	1
QCare ICU	Critical Care Company (C3)	1

### 3.) The level of integration between the available ICIS and the hospital information system

Integration with the hospital information system for administrative data exchange, the so-called admission, discharge and transfer (ADT) coupling, has been carried out in 83% of ICISs, and most systems are integrated with the electronic patient record and laboratory system as well. Direct integration with the radiology system and the pharmacy department on the one hand, and ICIS on the other hand is technically more demanding, resulting in an integration rate of only 25%.

### 4.) The effective use of highly detailed data extraction

The regular use of data extraction tools for management or scientific purposes is still limited (4 out of 12), and is only accomplished in university or tertiary non-academic referral hospitals.

### 5) The decision-making process in implementing an ICIS, including the benefits and obstacles perceived by the ICU decision makers

Data are obtained from the written 2005 questionnaire. The main anticipated benefits of an ICIS according to ICU directors are listed in order of importance in Table 3. The main concerns of buying an ICIS are listed in order of importance in Table 4. These major drawbacks are the cost, the need for dedicated personnel, and problems of integration with other hospital information systems.

**Table 3:** Main anticipated benefits of switching from paper charting to an ICIS

1. Automatic compact archiving;
2. Improved exchange of information between the different caregivers;
3. More complete and automatic data acquisition;
4. Higher quality of care with prevention of errors, in the first place medication errors;
5. Automatic calculation of scores and support for coding (e.g. APACHE II, SOFA, SAPS, TISS);
6. Automatic reporting and automatic generation of discharge documents;
7. Data extraction possibilities.

APACHE II: Acute Physiology and Chronic Health Evaluation II; SOFA: Sequential Organ Failure Assessment; SAPS: simplified acute physiology score; TISS: Therapeutic Intervention Scoring System

**Table 4:** Main drawbacks to buying an ICIS

1. Financial cost for initial implementation, maintenance and upgrading;
2. The need for dedicated IT personnel for configuration and end-user training;
3. Integration with the hospital information system;
4. Reliability;
5. Confidentiality issues;
6. Need for infrastructure adaptations.

Almost 40% of ICU directors are convinced that investing in an ICIS should be a top priority for their ICU. However, around 80% of ICU directors doubt whether there is enough evidence to ensure the cost/benefit of an ICIS.

The major reason for not implementing an ICIS is the substantial financial cost. One hospital even invested in a new paper medical record recently. The extra cost per ICU bed for installing an ICIS ranged between 20,000 and 25,000 Euros, which is a substantial investment. Twenty-nine out of 31 respondents hold the opinion that the government should finance at least 40% of total costs, and 1 out of 3 hospitals even feel that governmental financial assistance should cover over 70% of the implementation cost. Some ICU directors in our study even suggest full reimbursement by the government.

## Discussion

To our knowledge, this is the first study evaluating the actual use of IT applications in the critical care environment in a European country, together with its evolution over time. We have evaluated both the adoption rate of hospital-wide IT-applications, which are part of the hospital information system, as well as the use of dedicated ICU IT applications (i.e. ICIS).

In our study, we found that laboratory results and radiology images are digitally available and are used in respectively 100% and 93.5% of the Flemish ICUs. But the use of IT for computerized requests of laboratory analyses and radiology investigations is still rare. Although few surveys have been conducted in this respect, we have noted substantial national variability regarding this issue [13-18]. In line with our results, Jha et al. also found a low adoption rate of 12% of U.S. ICUs using computerized requests for laboratory orders [17]. However, Lapinsky has found that 52% of Canadian ICUs use computerized laboratory and radiology requests [13]. The reason for this substantial difference is unknown. Regarding the computerized prescription of medication, we note that Flemish ICUs make more use of it (i.e. 41.3%) than Canadian (22%) or American ICUs (5 to 15%) [13-16]. The higher adoption rates of medication CPOE in contrast with the low adoption rates for laboratory and radiology CPOE may perhaps partly be attributed to the increasing number of publications showing that medication CPOE can improve care by reducing medication errors [19-26].

In Flanders, the ICIS adoption rate is currently 19% and this low rate correlates well with the rate mentioned in the Canadian report by Lapinsky et al.[13]. However, in the latter survey, only 7 out of 50 ICUs (14%) capture data directly from patient monitors, and merely 6% are connected to infusion pumps or ventilators [13]. Our second survey in 2008 showed that by the end of 2009 the ICIS adoption rate would have increased to 26.9%. However, these adoption rates can be an overestimation, as many ICUs intend to implement an ICIS, but fail to do so. Therefore, in August 2010, we contacted the 5 Flemish ICUs of our study once more. In the 2008 survey these ICUs had expressed their plans to implement an ICIS in the near future (i.e. before 2010). Only one out of these 5 ICUs delayed the project due to financial reasons, which gives an actual ICIS implementation rate in Flanders of 25.3% (16/63).

In line with the survey by Jha et al. the Flemish larger and teaching hospitals are the leading ICUs investing in an ICIS (see Figure 2) [17]. Possibly the innovative role of teaching centres, the more powerful financial possibilities and the interest in scientific research facilitated by data extraction, have influenced the ultimate decision to surmount the barriers to implementation. However, there is a remarkable gap between the initial enthusiasm in data extraction and the actual use of it, as only 4 out of 12 ICUs query and use refined data for management or research purposes. The specific expertise needed to perform complex database queries remains an important obstacle, despite the availability of commercial data extraction software packages which reduce the need for extensive knowledge of the Structured Query Language (SQL) and the exact relational database structure.

In contrast to the USA or the UK, there are no Flemish governmental financial incentives for the computerization of ICUs [27,28]. Yet this survey shows that especially the high cost associated with the purchase and implementation of an ICIS is the most important obstacle (see Table 4). This finding is supported by the general literature on this issue [13,17]. It is, nevertheless, clear that governments are highly interested in optimising the cost-efficiency of intensive care medicine and are hence interested in detailed data on resource use and outcome. This would allow them to shift to a form of performance-based financing in the future. Therefore, financial incentives for a complete computerization of ICUs could result in a win-win situation both for the ICUs and for the government.

In our survey, it became clear that ICISs are not always used to their full capacity. In fact, two Flemish centres which implemented an ICIS experienced major difficulties in linking their systems to the hospital information system. These problems created failure for automated charting in one hospital, and failure of the pharmacy linking in the other. Furthermore, at least 5 other hospitals admit that the implementation schedule of an ICIS has been significantly delayed due to integration problems with the hospital information system and/or pharmacy department.

There are several limitations to our study. First, the response rate of the 2005 survey was only 57.5%. Nevertheless, this is in accordance with other response rates of similar written questionnaires [13,17]. We should also note that there was probably an important responding bias to the initial written questionnaires in 2005, which appear to include among the respondents particularly ICUs which had already implemented an ICIS. Furthermore, the non-respondents in this first survey admitted later in the telephone survey of 2008 that they did not return the questionnaire especially because they were not computerized. This means that the perceived implementation rate of 16.1% in 2005 was clearly an overestimation, and was actually only 9.3% (i.e. 5 out of 54 ICUs). Therefore, we can draw the relevant conclusion that the ICIS implementation rate in Flemish ICUs doubled from 2005 to 2008 to the level of 19% and increased further in August 2010 to 25.3%.

Second, we did not use a validated questionnaire. This implies limited applicability for parallel follow-up study. Finally, the responses indicate self-reported IT implementation rather than direct observation.

## Conclusions

Nearly all ICUs in Flanders use hospital-wide available IT applications such as computerized laboratory and radiology displays, although the computerized request for laboratory and radiology is still an exception. Furthermore, the adoption level of medication CPOE and ICIS remains relatively low. The implementation rate of ICISs has nearly doubled over the last three years, and has the potential to increase to 50% by the end of 2011. Major obstacles to implement specialized IT solutions are the high initial costs and maintenance costs, the complexity of integrating ICISs with existing hospital-information systems, and the unclear return on investment.

## List of abbreviations

IT: Information Technology; ICU: Intensive Care Unit; PACS: Picture Archiving and Communication System; CPOE: Computerized Physician Order Entry; ICIS: Intensive Care Information System.

## Competing interests

The authors declare that they have no competing interests.

## Authors' contributions

KC has made substantial contributions to the original concept, coordination and design, acquisition of the data, analysis and interpretation of the data, and drafting of the manuscript. SV participated in the design and the coordination of the study, the acquisition and analysis of the data, and drafting the manuscript. CD participated in the design and coordination of the study. DB participated in the design and helped to draft the manuscript. KS, SVH and FDT participated in the design of the study, and have been involved in revising it thoroughly. JD made substantial contributions to the concept and design, analysis and interpretation of the data, and has revised the manuscript thoroughly. All authors have reviewed and approved the final manuscript.

## Pre-publication history

The pre-publication history for this paper can be accessed here:

http://www.biomedcentral.com/1472-6947/10/62/prepub

## References

[B1] Kohn L Corrigan J Donaldson M To Err is Human: Building a safer Health Care System 1999 Washington DC, National Academy Press 25077248

[B2] eHealth ERA team. eHealth priorities and strategies in European Countries http://ec.europa.eu/information_society/activities/health/docs/policy/ehealth-era-full-report.pdf

[B3] Cullen DJ Sweitzer BJ Bates DW Burdick E Edmondson A Leape LL Preventable adverse drug events in hospitalized patients: a comparative study of intensive care and general care units Crit Care Med 1997 25 1289 1297 10.1097/00003246-199708000-00014 9267940

[B4] Herout PM Erstad BL Medication errors involving continuously infused medications in a surgical intensive care unit Crit Care Med 2004 32 428 432 10.1097/01.CCM.0000108876.12846.B7 14758159

[B5] Donchin Y Gopher D Olin M Badihi Y Biesky M Sprung CL Pizov R Cotev S A look into the nature and causes of human errors in the intensive care unit Crit Care Med 1995 23 294 300 10.1097/00003246-199502000-00015 7867355

[B6] Andrews LB Stocking C Krizek T Gottlieb L Krizek C Vargish T Siegler M An alternative strategy for studying adverse events in medical care Lancet 1997 349 309 130 10.1016/S0140-6736(96)08268-2 9024373

[B7] Pronovost PJ Berenholtz SM Ngo K McDowell M Holzmeuller C Haraden C Resar R Rainey T Nolan T Dorman T Developing and pilot testing quality indicators in the intensive care unit J Crit Care 2003 18 145 155 10.1016/j.jcrc.2003.08.003 14595567

[B8] Bates DW Larizgoitia I Prasopa-Plaizier N Jha AK Research Priority Setting Working Group of the WHO World Alliance for Patient Safety Global priorities for patient safety research BMJ 2009 14 338 b1775 10.1136/bmj.b1775 19443552

[B9] Garrouste Orgeas M Timsit JF Vesin A Schwebel C Arnodo P Lefrant JY Souweine B Tabah A Charpentier J Gontier O Fieux F Mourvillier B Troché G Reignier J Dumay MF Azoulay E Reignier B Carlet J Soufir L Selected Medical Errors in the ICU: Results of the IATROREF Study (Parts I and II) Am J Respir Crit Care Med 2009 19875690 10.1164/rccm.200812-1820OC

[B10] Kelley MA Angus D Chalfin DB Crandall ED Ingbar D Johanson W Medina J Sessler CN Vender JS The critical care crisis in the United States: a report from the profession Chest 2004 125 1514 7 10.1378/chest.125.4.1514 15078767

[B11] Ewart GW Marcus L Gaba MM Bradner RH Medina JL Chandler EB The critical care medicine crisis: a call for federal action: a white paper from the critical care professional societies Chest 2004 125 1518 21 10.1378/chest.125.4.1518 15078768

[B12] Irwin RS Marcus L Lever A The critical care professional societies address the critical care crisis in the United States Chest 2004 125 1512 3 10.1378/chest.125.4.1512 15078766

[B13] Lapinsky SE Holt D Hallett D Abdolell M Adhikari N Survey of information technology in Intensive care units in Ontario, Canada BMC Med Inform Decis Mak 2008 8 5 10.1186/1472-6947-8-5 18218117 PMC2233621

[B14] Levy MM Computers in the ICU J Crit Care 2004 19 199 200 10.1016/j.jcrc.2004.10.002 15648034

[B15] Manjoney R Clinical Information System Market - an insider's view Journal of Critical Care 2004 19 215 220 10.1016/j.jcrc.2004.09.004 15648037

[B16] Amarasingham R Pronovost P Diener-West M Goeschel C Dorman T Thiemann D Powe N Measuring Clinical Information Technology in the ICU setting: application in a Quality Improvement Collaborative J Am Med Inform Assoc 2007 14 288 294 10.1197/jamia.M2262 17329726 PMC2244889

[B17] Jha AK DesRoches CM Campbell EG Donelan K Rao SR Ferris TG Shields A Rosenbaum S Blumenthal D Use of electronic health records in U.S. hospitals N Engl J Med 2009 360 1629 38 10.1056/NEJMsa0900592 19321858

[B18] Aarts J Koppel R Implementation of computerized physician order entry in seven countries Health Affairs 2008 28 404 414 10.1377/hlthaff.28.2.404 19275996

[B19] Colpaert K Decruyenaere J Computerized Physician Order Entry in Critical Care Best Pract Res Clin Anaesthesiol 2009 23 27 38 10.1016/j.bpa.2008.07.002 19449614

[B20] Colpaert K Claus B Somers A Vandewoude K Robays H Decruyenaere J Impact of computerized physician order entry on medication prescription errors in the intensive care unit: a controlled cross-sectional trial Crit Care 2006 10 R21 10.1186/cc3983 16469126 PMC1550814

[B21] Bates DW Teich JM Lee J Seger D Kuperman G Ma'Luf N Boyle D Leape L The impact of computerized physician order entry on medication error prevention J Am Med Inform Assoc 1999 6 313 21 10428004 10.1136/jamia.1999.00660313 PMC61372

[B22] Bates DW Leape LL Cullen DJ Laird N Peterson L Teich J Laird N Peterson A Teich M Burdick E Hickey M Kleefield S Shea B Vander Vliet M Seger D Effect of computerized physician order entry and a team intervention on prevention of serious medication errors JAMA 1998 280 1311 6 10.1001/jama.280.15.1311 9794308

[B23] Evans R Pestotnik S Classen D Clemmer T Weaver L Orme J Lloyd J Burke J A Computer-Assisted Management Programme for Antibiotics and Other Antiinfective Agents NEJM 1998 338 232 260 10.1056/NEJM199801223380406 9435330

[B24] Peterson J Kuperman G Shek C Patel M Avorn J Bates DW Guided prescription of psychotropic medications for geriatric in-patients Arch Intern Med 2005 165 802 7 10.1001/archinte.165.7.802 15824302

[B25] Shulman R Singer M Goldstone J Bellingan G Medication errors: a prospective cohort study of hand-written and computerised physician order entry in the intensive care unit Crit Care 2005 9 16 21 10.1186/cc3049 16277713 PMC1297620

[B26] Fraenkel D Cowie M Daley P Quality benefits of an intensive care clinical information system Crit Care Med 2003 31 120 5 10.1097/00003246-200301000-00019 12545004

[B27] Ewart G Marcus L Gaba M Bradner R Medina J Chandler E The Critical Care Medicine Crisis; a call for federal action: a white paper from the critical care professional societies Chest 2004 125 1518 1521 10.1378/chest.125.4.1518 15078768

[B28] National Programme for IT (NPfIT) in the NHS homepage http://www.connectingforhealth.nhs.uk/(last accessed 8 April 2010).

